# Evolution of Oleosin in Land Plants

**DOI:** 10.1371/journal.pone.0103806

**Published:** 2014-08-08

**Authors:** Yuan Fang, Rui-Liang Zhu, Brent D. Mishler

**Affiliations:** 1 School of Life Science, East China Normal University, Shanghai, China; 2 University and Jepson Herbaria, and Department of Integrative Biology, University of California, Berkeley, California, United State of America; Wuhan Botanical Garden, Chinese Academy of Sciences, Wuhan, China

## Abstract

Oleosins form a steric barrier surface on lipid droplets in cytoplasm, preventing them from contacting and coalescing with adjacent droplets. Oleosin genes have been detected in numerous plant species. However, the presence of oleosin genes in the most basally diverging lineage of land plants, liverworts, has not been reported previously. Thus we explored whether liverworts have an oleosin gene. In *Marchantia polymorpha* L., a thalloid liverwort, one predicted sequence was found that could encode oleosin, possessing the hallmark of oleosin, a proline knot (-PX_5_SPX_3_P-) motif. The phylogeny of the oleosin gene family in land plants was reconstructed based on both nucleotide and amino acid sequences of oleosins, from 31 representative species covering almost all the main lineages of land plants. Based on our phylogenetic trees, oleosin genes were classified into three groups: M-oleosins (defined here as a novel group distinct from the two previously known groups), low molecular weight isoform (L-oleosin), and high molecular weight isoform (H-oleosin), according to their amino-acid organization, phylogenetic relationships, expression tissues, and immunological characteristics. In liverworts, mosses, lycophytes, and gymnosperms, only M-oleosins have been described. In angiosperms, however, while this isoform remains and is highly expressed in the gametophyte pollen tube, two other isoforms also occur, L-oleosins and H-oleosins. Phylogenetic analyses suggest that the M-oleosin isoform is the precursor to the ancestor of L-oleosins and H-oleosins. The later two isoforms evolved by successive gene duplications in ancestral angiosperms. At the genomic level, most oleosins possess no introns. If introns are present, in both the L-isoform and the M-isoform a single intron inserts behind the central region, while in the H-isoform, a single intron is located at the 5′-terminus. This study fills a major gap in understanding functional gene evolution of oleosin in land plants, shedding new light on evolutionary transitions of lipid storage strategies.

## Introduction

It is important to understand the evolution of cellular components in relation to their function, including lipid storage. Virtually all cells in plants (and many other organisms) synthesize triacylglycerols (TAGs) and reserve them in intracellular lipid droplets [1], which probably exist in most eukaryotes and some prokaryotes [2]–[4]. In plants, lipid droplets are present in diverse cell types for nutrient storage, stress response, and other purposes [5], [6]. Lipid droplets usually have a matrix of TAGs covered by a single layer of phospholipids (PLs) in which the structural protein oleosins are embedded [5], [7]. Lipid droplets in seeds are usually called oil bodies [8]. However, the term “oil body” is best reserved specifically for an organelle that is unique to liverworts, bounded by a biological membrane and contains lipid globules lying in a proteinaceous matrix [9]–[12]. While the proteins of the liverwort oil body are little studied, the oleosins coating the lipid droplets of many plant seeds have been well researched, including in the moss *Physcomitrella patens* (Hedw.) Bruch & Schimp [13], and green algae *Chlamydomonas reinhardtii* PA Dang [8].

Oleosins are small proteins embedded in PLs, forming a steric barrier surface to maintain the lipid droplet structure in cytoplasm [7], [14]. Oleosins stabilize lipid droplets as small entities and, at the same time, provide a large surface area per unit TAG. Oleosins facilitate lipase binding and lipolysis during germination due to the quick, easy, and economical conversion of the TAGs into free fatty acids via lipase mediated hydrolysis at the lipid droplet surface [15], [16]. Oleosin is the main protein on the lipid droplet surface, distinct from other lipid droplet associated proteins, caleosin and steroleosin [14], [17].

The anti-parallel β-stranded domain (ca. 72 residues) of oleosin penetrates the PLs into the core of the TAG matrix [18]. The central domain is a hydrophobic loop region, consisting of a proline knot motif possessing three proline residues and one serine residue (-PX_5_SPX_3_P-). This proline knot motif is highly conserved across all oleosin sequences so far identified, and is considered the hallmark for recognition of oleosin genes [19]. The hydrophobic domain is flanked by two other amphipathic domains residing on the organelle surface or partially embedded in the PLs. Both amphipathic domains are shorter than the central domain. The N-terminus domain is less conserved across oleosin sequences (in both length and sequence variation), while the C-terminus amphipathic α-helical domain is moderately conserved, especially within the same isoform [20].

Oleosins are small proteins of about 15 to 26 kDa in molecular weight [14], [21], [22], sequence length depends on the isoform and the plant species [15]. The insertion of 18 residues in the C-terminus domain defines two isoforms: high molecular weight isoform (H-oleosin, H-isoform) and low molecular weight isoform (L-oleosin, L-isoform). This C-terminal insertion accounts for the mass difference of 2 kDa between these two groups [20]. The H-class gene is more closely related to H-oleosins from other plant species than to L-oleosins within the same species, and vice versa [23]. Antibodies raised against L-oleosins do not cross-recognize H-oleosins, and those raised against H-class genes do not cross-recognize L-oleosins [24].

The expression of oleosin genes is tissue specific. Transcripts were detected in maturing seed, pollen, and tapetum, but always absent or with weak expression in vegetative tissues [13], [15], [25]. Kim et al [15] characterized oleosin genes in *Arabidopsis thaliana* (L.) Heynh. into three groups. The first group consisted of oleosins expressed solely in the seeds (S), the second expressed in the seeds and the floral microspores (SM), and the last group expressed in the floret tapetum (T).

In green algae (both chlorophytes and charophytes), besides oleosin-like proteins, the major lipid droplet protein (MLDP) has uniform expression in all cells, and was thought to prevent lipid droplets from aggregation [4], [6]. However, it has been recently reported that the MLDP is accumulated in ER subdomains and only partially wrapped around lipid droplets; *Spirogyra grevilleana* (Hassall) Kützing oleosin tagged with a Green Fluorescent Protein gene was observed enclosing *P. patens* lipid droplets, but this was hard to detect in algal tissue [8].

Oleosins are found in cytoplasm lipid droplets of almost all the green plants. However, the presence of oleosin in liverworts has not been reported previously. Oleosin genes have received considerable attention in recent years and have been studied in numerous plant species. The full-length DNA and/or cDNA encoding oleosins have been obtained from various species across green plants, including green algae [8], mosses [13], gymnosperms (pine [26]), and angiosperms, including *Arabidopsis* [15], [27]–[29], barley [30], castor bean [31], [32], coffee [25], cotton [33], maize [24], [34], olive [35], rapeseed [23], [36]–[40], sesame [41], and soybean [42]. Many oleosin genes are known, yet a comprehensive phylogenetic analysis of land plant oleosins based on both nucleotide and protein sequences, and intron insertion site among different isoforms, however, has not been previously reported.

This paper had dual goals. First, we explored whether liverworts, with their unique organelle, the oil body, have genes that encode either one or both of MLDP and oleosin. Second, we explored the evolution of the oleosin gene family including the representative that was detected in liverworts. As the sister group of all other land plants, liverworts occupy a strategic phylogenetic position for reconstructing one of the most important events in earth history, the conquest of land by plants. We furthermore explored the evolution of introns in the oleosin genes.

## Materials and Methods

### Data mining

Searches for predicted oleosin orthologs were performed using BLASTN and TBLASTN searches [43] in the Marchantiopsida Expressed Sequence Tag (EST) database at GenBank (http://ncbi.nlm.nih.gov/) using previously reported oleosin genes (see Table S1 online) as the query sequences. Oleosin cDNA sequences reported previously were also used as query sequences to search for oleosin predicted nucleotide sequences using the BLAST program (BLASTN) against *Citrus sinensis* (L.) Osbeck, *Glycine max* (L.) Merr., *Gossypium raimondii* Ulbr., *Oryza sativa* L., *P. patens*, *Populus trichocarpa* Torr. & Gray, *Ricinus communis* L., *Selaginella moellendorffii* Hieron, *Theobroma cacao* L., *Vitis vinifera* L., and *Zea mays* L. genomes in the Joint Genome Institute database (JGI, www.phytozome.net/search.php). In addition, oleosin nucleotide sequence of *Picea abies* (L.) Karst. and *Amborella trichopoda* Baill.were searched for in the Spruce Genome Project database (http://congenie.org/start) [44] and the *Amborella* Genome Database (http://www.amborella.org/) [45] respectively. In turn these nucleotide sequences were used as queries for data mining from the NCBI Trace Archive using BLAST against the *M. polymorpha* shotgun results. Oleosins and MLDPs in *M. polymorpha* were also searched for in JGI transcript assemblies by Sandra Floyd (pers. com.).

A similar search procedure was carried out in searching for homologs of MLDP in *M. polymorpha*. The accession numbers for these MLDP genes applied as queries in the present study are as follow: eight cDNA sequences, *Chlorella variabilis*_MLDP (EFN52470.1), *Coccomyxa* sp._MLDP (GW222322.1), *Dunaliella bardawil*_MLDP (JQ011391.1), *D. parva*_MLDP (JQ011392.1), *D. salina*_MLDP (JQ011390.1), *Haematococcus pluvialis*_MLDP (HQ213938.1), *Volvox carteri*_MLDP (XM002958607.1); two nucleotide sequences, *Chlamydomonas reinhardtii* oleosin chromosome9: 2981632–2984252, and *V. carteri* scaffold62: 48662–50757 in the JGI database.

### DNA extraction, PCR amplification, and sequencing

The material of *M. polymorpha* used in this research was collected from the greenhouse of the University of California Botanical Garden at Berkeley. A voucher specimen was deposited in UC/JEP (*Marchantia polymorpha*, USA, CA, Berkeley, University of California Botanical Garden at Berkeley, green house, on soil, *Y. Fang 20130226-1*). Total genomic DNA was extracted using the DNeasy Plant Minikit (QIAGEN). Polymerase chain reaction (PCR) was carried out using AccuPower PyroHotStart Taq PCR PreMix. Primer pairs for amplification of *M. polymorpha* oleosin are as followed, 5′-TTCAGTCCAATCTTGATACCTC-3′; C: 5′-ACTCGTGGGTAAAGGGCATG-3′. The temperature profile used for sequencing was 94°C for 5 min, then 30 cycles of 94°C for 20 sec, 20 sec at annealing temperature of 45°C, then 72°C for 45 sec, followed by a final extension step of 72°C for 5 min. PCR products were analyzed by electrophoresis in 2% agarose gels and detected by staining with ethidium bromide. PCR products of the correct size were cleaned and sequenced at the University of California, Berkeley, DNA Sequencing Facility.

### Phylogenetic analyses

Alignments were performed using ClustalW2 [46], [47] (default parameters for gap open penalty and gap extension penalty were used) for the oleosin protein sequences, then adjusted manually to optimize the alignment. Oleosin nucleotide sequences were aligned with MUSCLE [48], implemented through the Geneious package (version 6.1), using default settings (maximum number of iterations = 8; clustering method for later iterations: UPGMB).

Alignments were trimmed and exported for the phylogenetic analyses. We conducted phylogenetic analyses of both datasets using maximum likelihood implemented in RAxML-HPC2 version 7.4.4 on the CIPRES portal [49], [50]. One thousand replicates of rapid bootstrap analyses were performed using RAxML v7.4.4 (employing the GTRGAMMA model of evolution for tree inference, and GTRCAT model of evolution for the nucleic acid dataset; and the CAT model for the protein dataset; with 1,000 bootstrap replicates.).

## Results

### Data mining

No MLDP gene was found in the *Marchantia* EST database. Two *M. polymorpha* predicted oleosin fragments from shotgun results were found in the GenBank Trace Archive (GWBO52615.b1 [943 bps] and GWBO44779.b1 [833 bps]). Both sequences encode partial amino acid sequences of an oleosin gene. GWBO52615.b1 translated into a 88 amino acid-long sequence, lacking the proline knot motif. It begins immediately after the loop region, and its translated amino acid sequence is conserved to the left central domain (the part behind the proline knot motif). The length of GWBO44779.b1 encoding region is 148 amino acids, lacking the starting methionine, but possessing the central domain with the central proline knot motif. One oleosin encoding region located on *M. polymorpha* scaffold_00105 was found containing a long open reading frame (ORF) without introns. The deduced sequence is 160 amino acids in length.

In amino acid sequence comparison, the translated amino acid sequences of GWBO52615.b1 were 47.83% identical to the *Physcomitrella patens*_OLE3 and 47.56% identical to the *Pinus taeda*_OLE. The translated amino acid sequences of GWBO44779.b1 were 27.03% identical to the *Physcomitrella patens*_OLE3, and 27.27% identical to the *Selaginella moellendorffii*_OLE8. The deduced amino acid sequence of the full-length *Marchantia* oleosin on Scaffold_00105 was 46.32% identical to *Selaginella moellendorffii*_OLE8 and 39.87% identical to *Physcomitrella patens*_OLE1. The *Marchantia* oleosin-encoding region on Scaffold_00150 shared up to 97.28% sequence identity with GWBO44799.b1 and 98.10% sequence identity with GWBO52615.b1. The two fragments showed 93.79% sequence identity. Mismatches only occurred at the termini and were probably due to sequencing error. This suggests that the two predicted *Marchantia* oleosin fragments in the Trace Archive were likely from the same locus, the oleosin encoding region found on scaffold_00105.

### DNA extraction, PCR amplification and sequencing

**Figure 1:**
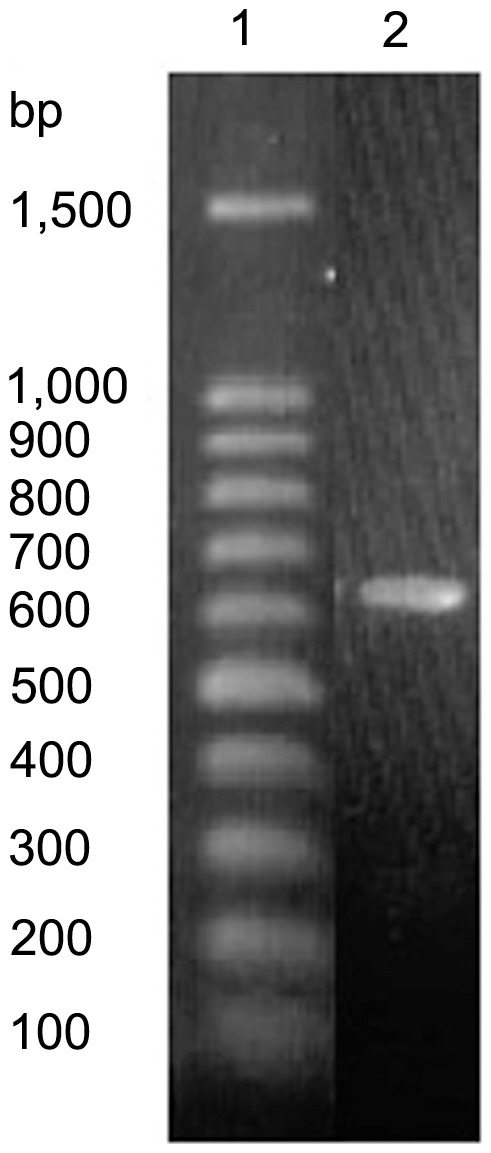
PCR results of *Marchantia polymorpha* oleosin. 1, molecular weight marker; 2, *M. polymorpha* oleosin.

**Figure 2:**
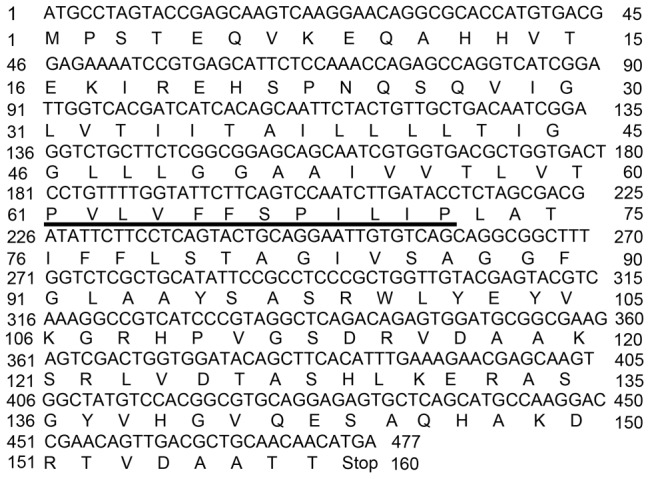
Nucleotide and translated amino acid sequence of *Marchantia polymorpha* oleosin DNA. The 12 residue proline knot motif is shown underlined.

**Figure 3:**
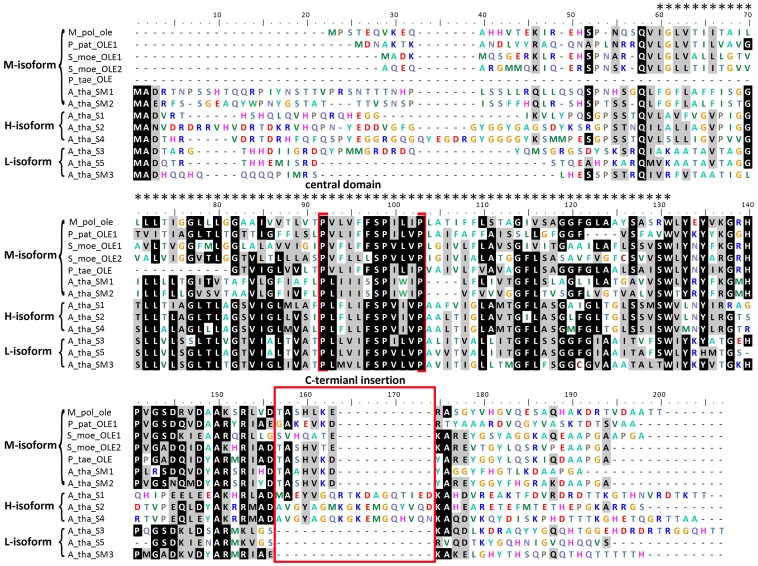
Complete amino acid sequence comparison of three oleosin isoforms from diverse species. The alignments were generated with the clustalW2 program and then adjusted manually to optimize the alignment. Residues with the highest conservation are in black, while similar residues are shaded in gray. Oleosin isoforms are indicated on the right side. Dots above the sequences display the central hydrophobic region (72 amino acids). The four invariable residues of the proline knot sequence (-PX_5_SPX_3_P-) are highlighted with brackets. The 18-amino acid insertions present in the C-terminus domain both in M-oleosins and H-oleosins are boxed. Unaligned residues are shown as dashes within the sequence. Abbreviations: M_pol: *Marchantia polymorpha*; P_pat: *Physcomitrella patens*; S_moe: *Selaginella moellendorffii*; P_tae: *Pinus taeda*; A_tha: *Arabidopsis thaliana*.

A *M. polymorpha* oleosin was cloned successfully with a continuous ORF (Figure 1). The ORF was 477 nucleotides long and encoded a peptide having 160 residues (Figure 2). When aligned to the sequences of other oleosins the highest similarity was located on the central domain while the N and C-termini was less conserved (Figure 3).

### Phylogenetic analyses

**Figure 4:**
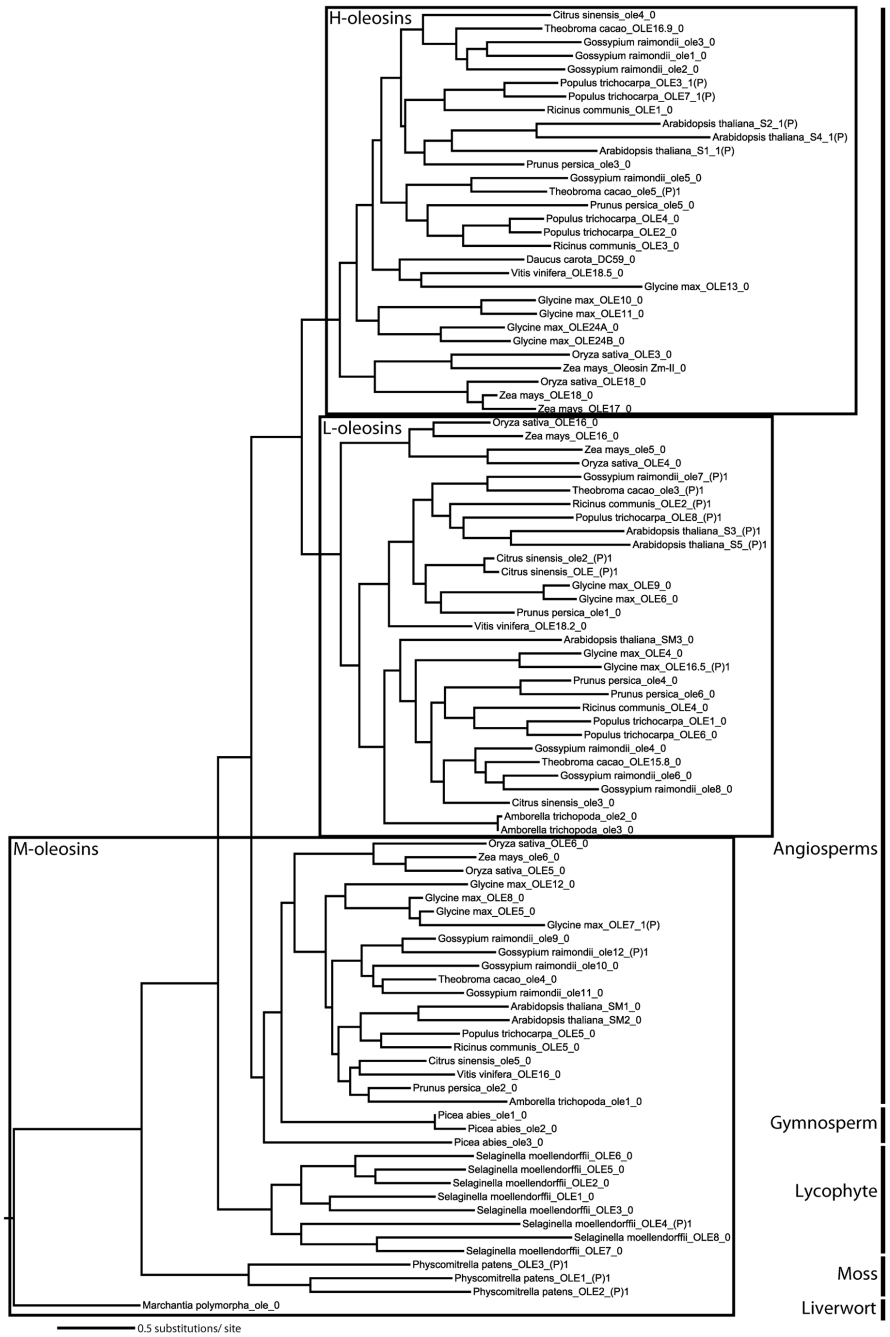
Oleosin phylogeny in land plants inferred by maximum likelihood analysis of nucleotide sequences. The topology is rooted by *Marchantia polymorpha* representing the most basally diverging lineage of land plants, liverworts. The three oleosin isoforms are framed separately. The information followed the second underscore in each terminal node shows the position of intron in oleosin nucleotide sequences. Abbreviations used in Figures 4, 5 and 7 are as follows: OLE, published or submitted oleosin genes in GenBank (For more information, see Table S1 online); ole (lower case), predicted oleosin genes from sequenced species in Joint Genome Institute database in this study; 0 (the number behind the second underscore in terminal node), no intron insertion in encoding region; 1(P), the site of intron insertion before the central domain coding region; (P)1, the site of intron insertion after the central domain coding region.

**Figure 5:**
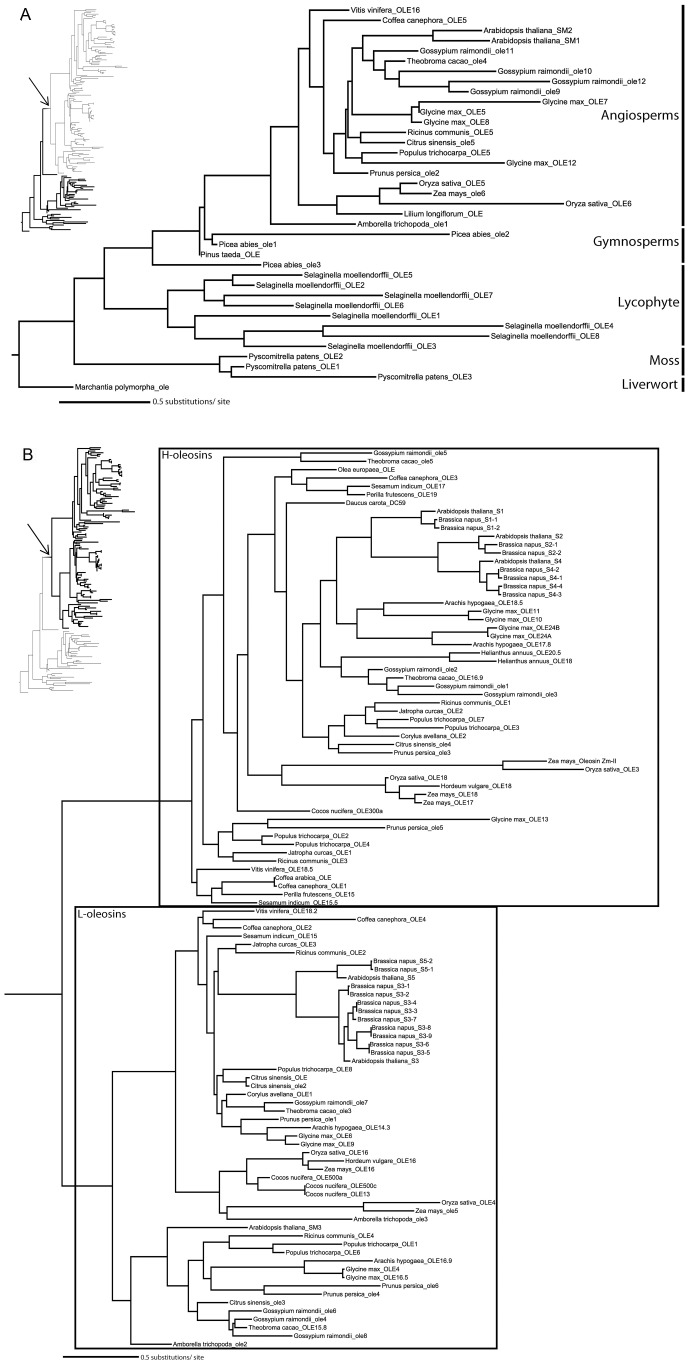
Two subtrees of the maximum-likelihood (ML) tree of oleosins in land plants inferred from a protein alignment (ClustalW2) by analysis. (A) M-oleosins; (B) L-oleosins and H-oleosins. The parts of phylogenetic tree shown are highlighted in the thumbnail trees at left. The arrow represents where the clade shown in Figure 5B subtree attaches. The topology is rooted by *Marchantia polymorpha* representing the most basally diverging lineage of land plants, liverworts. The L-oleosin and H-oleosin isoforms are framed in Figure 5B.

**Figure 6:**
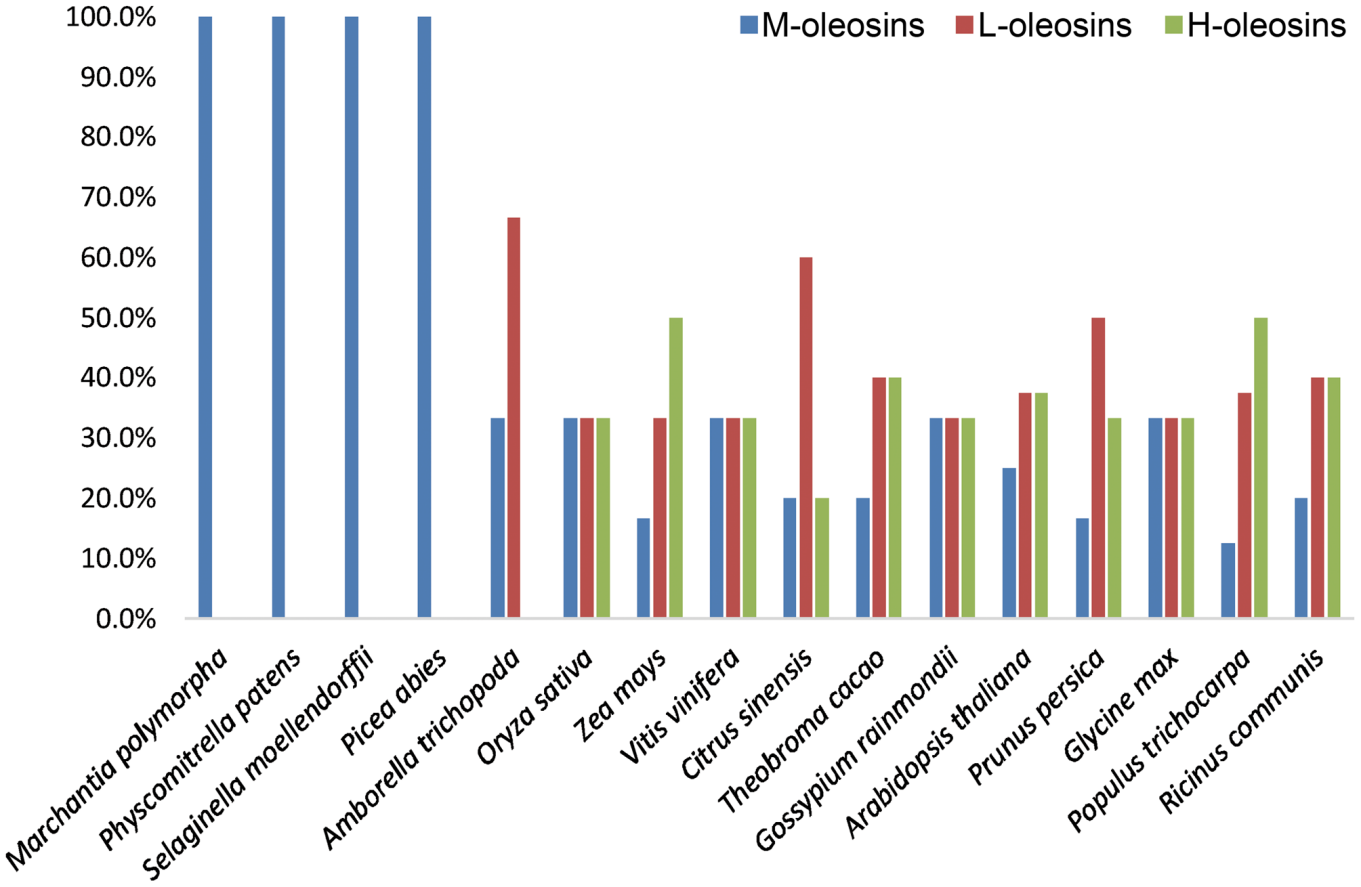
The model transition of the three oleosin isoform distributions (percentage) in genomes of land plants throughout evolution.

The newly obtained liverwort oleosin gene allowed us to perform the first detailed phylogenetic analysis of oleosin gene evolution including the most basally diverging lineage of land plants. The separate protein and nucleotide analyses yielded similar tree topologies with M-oleosins, L-oleosins, and H-oleosins forming three distinct groups (Figure 4 and 5). There appears to have been only one oleosin gene in the common ancestor of vascular plants, because in *Marchantia*, *Physcomitrella*, and *Selaginella*, oleosin genes within the same species form a clade indicating proliferation of gene families following their divergence. However, H-oleosins and L-oleosins coexist in monocots and eudicots along with the maintenance of M-isoforms (Figure 6). No H-oleosin gene was found in current available *Amborella* genome, which possesses the other two oleosin isoforms. Thus, there appears to have been at least two oleosin genes in the common ancestor of angiosperms, with subsequent proliferation of additional copies in some lineages.

In addition to the two isoform classes previously known (H-isoform, L-isoform), we discovered a new class of oleosins that we name M-isoform. The existence of this distinct class is due to results of our phylogenetic analyses (Figure 4 and 5) and their special C-terminus insertion (Figure 3). The molecular weight of M-isoform falls in between that of L-isoform and H-isoform, and is named following the principle of L-isoform and H-isoform nomenclature.

## Discussion

The central hydrophobic domain is highly conserved across all isoforms. The central domain forms a loop region, penetrating through the PL membrane into the TAG matrix. The sequence alignment shows that the difference among the three oleosin isoforms is consistently in the C-terminus (Figure 3). There is an insertion of about seven residues in M-oleosins, a loss of residues in L-oleosins, and an insertion of approximately 18 residues in H-oleosins. This suggests that the variation in C-terminus amphipathic domain may be related to constructing a more efficient location for lipase attachment or for organelle interaction with glyoxysomes during seed germination and postgerminative growth of seedlings [20]. Based on the insertion of seven residues in the C-terminus domain, and according to its phylogenetic relationship, the *Marchantia* oleosin gene should be grouped into the M-isoform class.

The *Coffea canephora*_OLE5 was considered as a H-isoform oleosin in previous reports because of a likely 18 residue insertion in its C-terminal domain [25]. However, in our analysis, it clustered with M-oleosins in the phylogenies. In addition, according to our alignment the C-terminus of the *Coffea canephora*_OLE5 gene does not possess a traditional 18 residue insertion at the C-terminal region characteristic of H-oleosins, but rather possesses the DAYR repeat in C-terminus as in *Arabidopsis thaliana*_SM1 and SM2, which belong to the M-oleosins. Thus we suggest that the *Coffea canephora*_OLE5 should be classified as an M-oleosin, based on its sequence structure and phylogenetic relationship.

**Figure 7:**
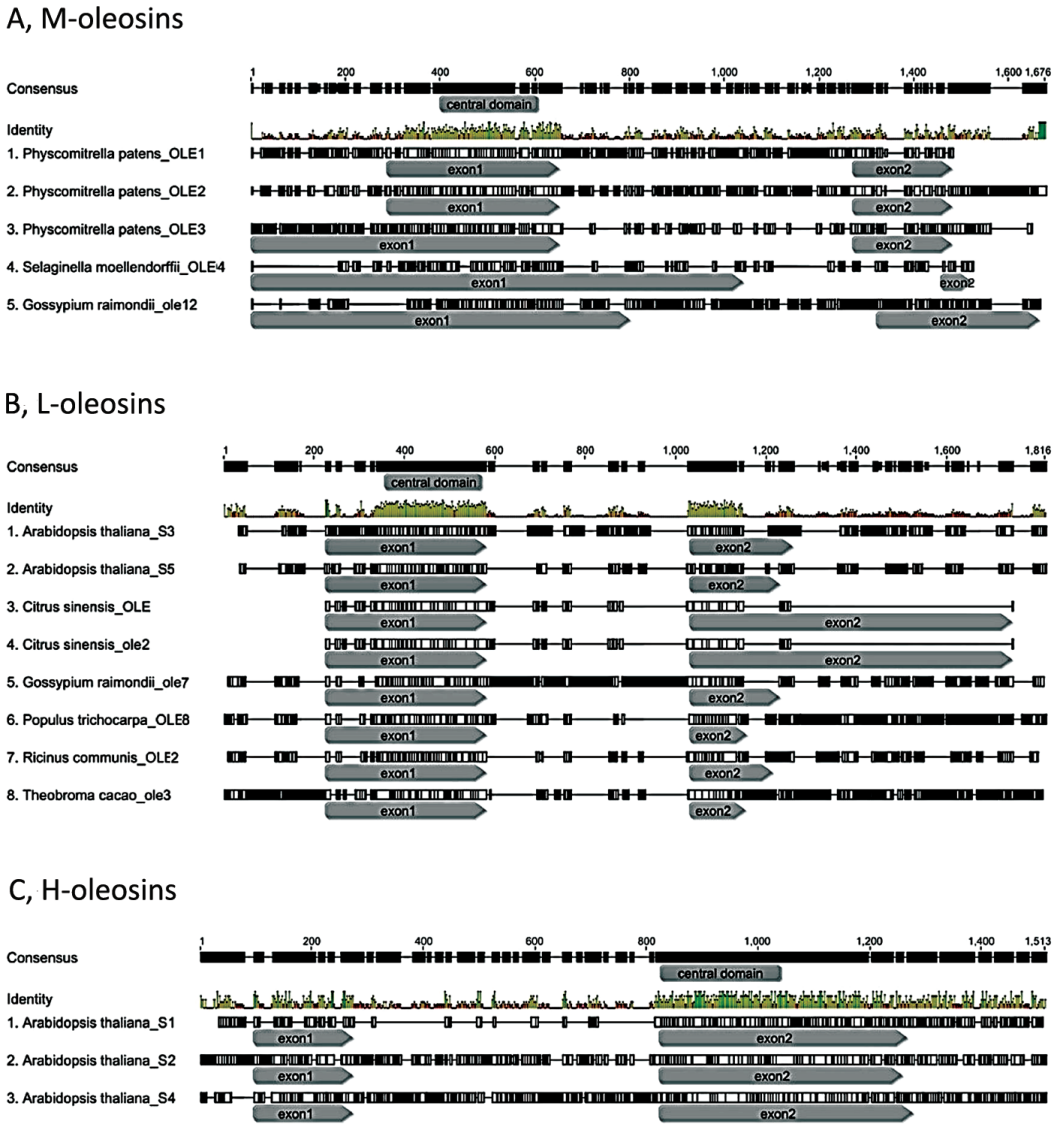
The intron position in each of the three oleosin isoforms. At the nucleotide level, Joint Genome Institute database predict that most of oleosins possess no introns, whereas oleosin genes with intron insertion sites a single intron preceding or following the sequence encoding the central domain. The intron insertion sites are variable in oleosins, but nearly conserved within each isoform. The region of the central domain is labeled in the ‘consensus’ row. The intron positions are in between of the two encoding regions: exon1 and exon2. (A) M-oleosins; (B) L-oleosins; (C) H-oleosins.

It appears that most of the oleosins possess no introns, whereas oleosin genes with intron insertion sites contain a single intron preceding or following the sequence encoding the central domain. Though nucleotide sequences of introns are different from each other, all are U2-type splice GT-AG introns [51]–[53]. The intron insertion sites are variable across oleosins, but are almost conserved within each isoform (Figure 4 and 7). It is interesting to note that no intron was predicted in the region encoding the central domain. The introns are located at 3′-terminus in both the M-isoform and the L-isoform, while the intron inserts in 5′-terminus in the H-isoform. In M-oleosins (Figure 7A), the position of the intron is conserved among the three *Physcomitrella* oleosins at the middle of the 3′-terminus region, while those in *Gossypium raimondii*_ole12 and *Selaginella moellendorffii*_OLE4 are located almost near the end of the 3′-terminus region. Among L-oleosins the position of a single intron (except the *Glycine max*_OLE16.5) in each of the eight genes is conserved, inserting just at the connection of the central region and 3′-terminus (Figure 7B). The site of the intron is almost always located in the 5′-terminus for H-oleosins. In *Arabidopsis*, the intron position is immediately before the central region (Figure 7C), while those in *Populus* are located at the very beginning of 5′-terminus regions. Two exceptions were identified in this analysis of nucleotide sequences. They are *Glycine max_*ole7, which has an intron insertion at the very beginning of the 5′-terminus region, deviating from the majority pattern in M-oleosins; and *Theobroma cacao*_ole5, whose intron insertion site is near the middle of 3′-terminus region in nucleotide sequence, although due to the 18 amino acid insertion in its C-terminus, it should be grouped into the H-isoforms. The intron insertion sites in M-oleosins are more variable than those in L-oleosins and H-oleosins. The intron insertion position is conserved in eight out of nine L-oleosins, suggesting that it had inserted in the encoding region early in the evolution of this gene lineage. On the other hand, because the intron insertion positions in H-oleosins are conserved only within species it is likely that those introns were inserted independently.

In earlier-diverging lineages of land plants, including liverworts, mosses, lycophytes, and gymnosperms, only M-oleosins have been identified. In angiosperms, M-oleosins remain and are expressed in the angiosperm gametophyte and pollen tube. The *Arabidopsis* pollen oleosins (*Arabidopsis thaliana*_SM1 and SM2) [15] and the putative rice pollen oleosin (*Oryza sativa*_OLE5) [55] all cluster in the class of M-oleosins. Pollen intracellular lipid droplets and membranes are primarily under the control of the gametophytic genome [54]. Jiang et al [55] confirmed that oleosin isoforms are not cross-recognized, using immunological comparisons of lily pollen oleosin and two classes of sesame seed oleosins.

Combined with the evidence from gene structure, intron insertion positions, tissue expression, and immunological characteristics, our phylogenetic analyses confirm that in earlier-diverging lineages of land plants, including liverworts, mosses, lycophytes, and gymnosperms, only M-oleosins are found. On the other hand, three isoforms were identified in most monocots and eudicots. This finding leads to the inference that M-oleosin is the most primitive oleosin isoform among the three. H-oleosin and L-oleosin probably derived from a secondary duplication after an initial duplication event that gave rise to their common ancestor. The two successive duplication events happened after the origin of angiosperms but before the divergence of monocots and eudicots. Since the H-oleosin isoform has not been found in current available *Amborella* genome, the completion of further early-diverging angiosperm genomes may make the evolution of H-oleosin and L-oleosin clearer.

The life cycle of “bryophytes” is gametophyte-dominant [56]–[59]. The gametophyte phase persists in angiosperms and interestingly M-oleosins are expressed in pollen lipid droplets [15], [55]. Within seed plants, gene duplications and rearrangement events resulted in new isoforms expressed in the diploid phase. In previous studies, lipid droplets have been reconstituted artificially with TAGs, PLs, and oleosins. Results in sesame showed the lipid droplets could be stabilized by both L- and H-isoforms, but L-oleosin gave slightly more structural stability than H-oleosin [20], [40]. Whether M-oleosin provides similar structural stability of lipid droplets to H-oleosin or L-oleosin remains to be tested.

In order to gain a better understanding of oleosin gene evolution in land plants, it will be important for complete genomes to become available for key clades such as hornworts, ferns, and gymnosperms. Detailed cloning studies and phylogenetic analyses of oleosin genes within liverworts will be of considerable interest as well, because this clade possesses a special organelle, the oil body. The liverwort oil body is thought to be unique, and a likely synapomorphy uniting them, thus further investigation in liverworts of genes known to be functionally involved in lipid droplets in other plants is important.

## Supporting Information

Table S1 Characteristics of 145 oleosins in land plants.
(DOC)
