# Supplementary material for: Evolution of Oleosin in Land Plants
Source: PLoS One. 2014 Aug 8;9(8):e103806. doi: 10.1371/journal.pone.0103806 (PMC4126676; doi:10.1371/journal.pone.0103806)
Supplement: Table S1 — Characteristics of 145 oleosins in land plants. (DOC) [file pone.0103806.s001.doc]

| **Table S1**: Characteristics of 145 oleosins in land plants. | | | | | |
| --- | --- | --- | --- | --- | --- |
| Species | Name | GeneID on JGI databse | Protein (GenBank accession number) | Isoform | Reference |
| *Arabidopsis thaliana* (arabidopsis) | S1 | At3g01570 | AAF01542 | H | Kim et al 2002 |
|  | S2 | At3g27660 | BAB02690 | H |  |
|  | S3 | At4g25140 | CAB36756 | L |  |
|  | S4 | At5g40420 | BAB11599 | H |  |
|  | S5 | At5g51210 | BAAQ7384 | L |  |
|  | SM1 | At1g48990 | AAF69712 | M |  |
|  | SM2 | At3g18570 | BAB02215 | M |  |
|  | SM3 | At2g25890 | AAC42242 | L |  |
| *Brassica napus* (rapeseed) | S1-1 |  | ACG69504.1 | H | Jolivet et al 2009 |
|  | S1-2 |  | ACG69505.1 | H |  |
|  | S2-1 |  | ACG69503.1 | H |  |
|  | S2-2 |  | ACG69506.1 | H |  |
|  | S3-1 |  | ACG69513.1 | L |  |
|  | S3-2 |  | ACG69514.1 | L |  |
|  | S3-3 |  | ACG69515.1 | L |  |
|  | S3-4 |  | ACG69516.1 | L |  |
|  | S3-5 |  | ACG69517.1 | L |  |
|  | S3-6 |  | ACG69518.1 | L |  |
|  | S3-7 |  | ACG69519.1 | L |  |
|  | S3-8 |  | ACG69520.1 | L |  |
|  | S3-9 |  | ACG69521.1 | L |  |
|  | S4-1 |  | ACG69507.1 | H |  |
|  | S4-2 |  | ACG69508.1 | H |  |
|  | S4-3 |  | ACG69509.1 | H |  |
|  | S4-4 |  | ACG69510.1 | H |  |
|  | S5-1 |  | ACG69511.1 | L |  |
|  | S5-2 |  | ACG69512.1 | L |  |
| *Zea mays* (maize) | OLE16 | GRMZM2G337229 | AAA68065.1 | L | Qu et al 1990, Lee & Huang 1994 |
|  | OLE17 | GRMZM2G480954 | AAA68066.1 | H |  |
|  | OLE18 | AC206941.2_FG002 | AAA67699.1 | H |  |
|  | Oleosin Zm-II | GRMZM2G410152 | AFW86598.1 | H | Schnable et al 2009 |
|  | ole5 | GRMZM2G333069 |  | L | Predicted in this study |
|  | ole6 | GRMZM2G096435 |  | M |  |
| *Arachis hypogaea* (peanut) | OLE17.8 |  | ABS28870.1 | H | Li et al 2009 |
|  | OLE18.5 |  | ABS28871.1 | H |  |
| *Arachis hypogaea* (peanut) | OLE14.3 |  | AAZ20277.1 | L | UP |
|  | OLE16.5 |  | AAU21501.1 | L | Pons et al 1998 |
| *Sesamum indicum* (sesame) | OLE15 |  | AAD42942.1 | L | Tai et al 2002 |
|  | OLE15.5 |  | AAB58402.1 | H | Chen et al 1997 |
|  | OLE17 |  | AAG23840.1 | H | Tai et al 2002 |
| Species | Name | GeneID on JGI databse | Protein (GenBank accession number) | Isoform | Reference |
| *Cocos nucifera* (coconut) | OLE500a |  | ACH91012.1 | L | Regalado et al 2008 |
|  | OLE500c |  | ACH91013.1 | L |  |
|  | OLE300a |  | ACH91011.1 | L |  |
|  | OLE13 |  |  |  | Li & Fan 2009 |
| *Lilium longiflorum* (lily) | OLE |  | ABK40507.1 | M | Jiang et al 2007 |
| *Coffea canephora* (coffee) | OLE1 |  | AAX49389.1 | H | Simkin et al 2006 |
|  | OLE2 |  | AAX49390.1 | L |  |
|  | OLE3 |  | AAX49391.1 | H |  |
|  | OLE4 |  | AAX49392.1 | L |  |
|  | OLE5 |  | AAX49393.1 | H |  |
| *Coffea arabica* (coffee) | OLE |  | AAY14574.1 | H | Simkin et al 2006 |
| *Theobroma cacao* (cacao) | OLE15.8 | 1EG037237 | AAM46778.1 | L | Guilloteau et al 2003 |
|  | OLE16.9 | 1EG016949 | AAM46777.1 | H |  |
|  | ole3 | 1EG014844 |  | L | Predicted in this study |
|  | ole4 | 1EG012246 |  | M |  |
|  | ole5 | 1EG012191 |  | H |  |
| *Daucus carota* (carrot) | DC59 | S47635.1 | Q43123 | H | Hatzopoulos et al 1990 |
| *Gossypium raimondii* (cotton) | ole1 | 006G033000 |  | H | Predicted in this study |
|  | ole2 | 001G264500 |  | H |  |
|  | ole3 | 011G094900 |  | H |  |
|  | ole4 | 010G103700 |  | L |  |
|  | ole5 | 009G267600 |  | H |  |
|  | ole6 | 009G047800 |  | L |  |
|  | ole7 | 001G051400 |  | L |  |
|  | ole8 | 001G024800 |  | L |  |
|  | ole9 | 007G007900 |  | M |  |
|  | ole10 | 006G063600 |  | M |  |
|  | ole11 | 008G285100 |  | M |  |
|  | ole12 | 002G174000 |  | M |  |
| *Helianthus annuus* (sunflower) | OLE20.5 |  | CAA44224.1 | H | Cummins & Murphy 1992 |
|  | OLE18 |  | CAA55348.1 | H | Thoyts et al 1995 |
| *Glycine max* (soybean) | OLE24A | Glyma19g13060 | AAA17854.1 | H | Kalinski et al 1991 Sarmiento et al 1997 |
|  | OLE24B | Glyma16g07800 | AAA17855.1 | H |  |
|  | OLE16.5 | Glyma04g08220 | NP_001236098.1 | L | UP |
|  | OLE4 | Glyma06g08290.2 | XP_003526464.1 | L | UP (NCBI Annotation) |
|  | OLE5 | Glyma05g07880 | XP_003524510.1 | M |  |
|  | OLE6 | Glyma10g33760 | NP_001237482.1 | L |  |
|  | OLE7 | Glyma14g15015 | XP_003545490.1 | M |  |
|  | OLE8 | Glyma17g13120 | XP_003549810.1 | M |  |
| Species | Name | GeneID on JGI databse | Protein (GenBank accession number) | Isoform | Reference |
| *Glycine max* (soybean) | OLE9 | Glyma20g33850 | XP_003556321.1 | L | UP (NCBI Annotation) |
|  | OLE10 | Glyma05g08880 | XP_003524555.1 | H |  |
|  | OLE11 | Glyma19g00400 | XP_003554272.1 | H |  |
|  | OLE12 | Glyma06g23340 | XP_003527133.1 | M |  |
|  | OLE13 | Glyma17g09390 | XP_003549608.1 | H |  |
| *Oryza sativa* (rice) | OLE18 | Os03g49190.1 | AAC02240.1 | H | Chen et al 1996 |
|  | OLE16 | Os04g46200.1 | AAC02239.1 | L |  |
|  | OLE3 | Os06g27910.1 | NP_001057629.1 | H | Tanaka et al 2008 |
|  | OLE4 | Os09g15520 | NP_001062874.1 | L |  |
|  | OLE5 | Os05g50110 | NP_001056403.1 | M |  |
|  | OLE6 | Os01g45624 | NP_001043695.1 | M |  |
| *Physcomitrella patens* (moss) | OLE1 | Pp1s84_138V6.1 | EDQ68725.1 | M | Huang et al 2009 |
|  | OLE2 | Pp1s21_351V6.1 | EDQ79003.1 | M |  |
|  | OLE3 | Pp1s180_16V6.1 | EDQ60469.1 | M |  |
| *Selaginella moellendorffii* (club moss) | OLE1 | scaffold_1000273 | EFJ36766.1 | M | Banks et al 2011 |
|  | OLE2 | gw1.6.504.1 | EFJ27139.1 | M |  |
|  | OLE3 | e_gw1.6.1478.1 | EFJ27356.1 | M |  |
|  | OLE4 | scaffold_7000458 | EFJ32739.1 | M |  |
|  | OLE5 | e_gw1.17.244.1 | EFJ27358.1 | M |  |
|  | OLE6 | gw1.27.92.1 | EFJ11452.1 | M |  |
|  | OLE7 | gw1.14.303.1 | EFJ20479.1 | M |  |
|  | OLE8 | gw1.46.99.1 | EFJ18108.1 | M |  |
| *Populus trichocarpa* (cotton wood) | OLE1 | 018G057800 | EEF07326.1 | L | Tuskan et al 2006 |
|  | OLE2 | 015G082100 | EEF06313.1 | H |  |
|  | OLE3 | 017G071800 | EEF03937.1 | H |  |
|  | OLE4 | 012G083400 | EEE96879.1 | H |  |
|  | OLE5 | 012G059400 | EEE96148.1 | M |  |
|  | OLE6 | 006G234900 | EEE93050.1 | L |  |
|  | OLE7 | 001G345800 | EEE84854.1 | H |  |
|  | OLE8 | 001G080000 | EEE82732.1 | L |  |
| *Pinus taeda* (pine)  *Hordeum vulgare* (barley) | OLE |  | FJ094810.2 | L | Lee et al 1994 |
|  | OLE18 |  | CAA57994 | H | Aalen 1995 |
|  | OLE16 |  | CAA57995 | L |  |
| *Ricinus communis* (castor bean) | OLE1 | 29917.m001992 | AAR15171.1 | H | Eastmond 2004 |
|  | OLE2 | 30147.m014333 | AAR15172.1 | L |  |
|  | OLE3 | 30147.m013891 | XP_002511342.1 | H | UP |
|  | OLE4 | 29794.m003372 | XP_002516493.1 | L |  |
|  | OLE5 | 30174.m008728 | XP_002511984.1 | M |  |
| *Prunus persica* (almond) | ole1 | ppa012948m.g |  | L | Predicted in this study |
|  | ole2 | ppa012323m.g |  | M |  |
|  | ole3 | ppa012747m.g |  | H |  |
|  | ole4 | ppa018784m.g |  | L |  |
| Species | Name | GeneID on JGI databse | Protein (GenBank accession number) | Isoform | Reference |
| *Prunus persica* (almond) | ole5 | ppa011111m.g |  | H | Predicted in this study |
|  | ole6 | ppa025868m.g |  | L |  |
| *Corylus avellana* (filbert) | OLE1 |  | AAO65960 | L | UP |
|  | OLE2 |  | AAO67349 | H |  |
| *Citrus sinensis* (orange) | OLE | 1g040357m.g | CAA88360.1 | L | Naot et al 1995 |
|  | ole2 | 1g041282m.g |  | L | Predicted in this study |
|  | ole3 | 1g045010m.g |  | L |  |
|  | ole4 | 1g041202m.g |  | H |  |
|  | ole5 | 1g047028m.g |  | M |  |
| *Olea europaea* (olive) | OLE |  | AAL92479 | H | Giannoulia et al 2007 |
| *Perilla frutescens* (perilla) | OLE15 |  | AAG43516 | H | UP |
|  | OLE19 |  | AAG24455 | H |  |
| *Jatropha curcas* (jatropha) | OLE1 |  | ABW90148.2 | H | Popluechai et al 2010 |
|  | OLE2 |  | ABW90149.2 | H |  |
|  | OLE3 |  | ABW90150.2 | L |  |
| *Vitis vinifera* (grape) | OLE16 | GSVIVT01008228001 | XP_002273242.1 | M | UP (NCBI Annotation) |
|  | OLE18.2 |  | XP_002281381.1 | H |  |
|  | OLE18.5 |  | XP_002281746.1 | L |  |
| *Picea abies* (spruce) | ole1 | MA_79152 |  | M | Predicted in this study (from Spruce Genome Project) |
|  | ole2 | MA_853685 |  | M |  |
|  | ole3 | MA_393573 |  | M |  |
| *Amborella trichopoda* (amborella) | ole1 | lcl|evm_27.model.AmTr_v1.0_scaffold00147.30 |  | M | Predicted in this study (from *Amborella* Genome Database) |
|  | ole2 | lcl\|evm_27.model.AmTr_v1.0_scaffold00056.169 |  | L |  |
|  | ole3 | lcl\|evm_27.model.AmTr_v1.0_scaffold00111.94 |  | L |  |
| Note: nomenclature of the published or submitted oleosins on Joint Genome Institute (JGI) database and GenBank follows the corresponding reference, except the *Picea abies* oleosins are from Spruce Genome Project database, and the *Amborella trihopoda* oleosins are from *Amborella* Genome Database. Highly simplified names for NCBI Annotation and predicted genes are used in this report solely for clarity in presentation.  Abbreviations: OLE = published or submitted oleosins on GenBank; ole (lower case) = predicted oleosin genes from sequenced species in JGI database in this study; UP =unpublished data; H = H-oleosin; L = L-oleosin; M = M-oleosin. | | | | | |
